# NTTMUNSW BioC modules for recognizing and normalizing species and gene/protein mentions

**DOI:** 10.1093/database/baw111

**Published:** 2016-07-27

**Authors:** Hong-Jie Dai, Onkar Singh, Jitendra Jonnagaddala, Emily Chia-Yu Su

**Affiliations:** ^1^Department of Computer Science and Information Engineering, National Taitung University, Taitung, Taiwan; ^2^Interdisciplinary Program of Green and Information Technology, National Taitung University, Taitung, Taiwan; ^3^Graduate Institute of Biomedical Informatics, College of Medical Science and Technology, Taipei Medical University, Taipei, Taiwan; ^4^School of Public Health and Community Medicine, the University of New South Wales, Sydney, Australia; ^5^Prince of Wales Clinical School, the University of New South Wales, Australia

## Abstract

In recent years, the number of published biomedical articles has increased as researchers have focused on biological domains to investigate the functions of biological objects, such as genes and proteins. However, the ambiguous nature of genes and their products have rendered the literature more complex for readers and curators of molecular interaction databases. To address this challenge, a normalization technique that can link variants of biological objects to a single, standardized form was applied. In this work, we developed a species normalization module, which recognizes species names and normalizes them to NCBI Taxonomy IDs. Unlike most previous work, which ignored the prefix of a gene name that represents an abbreviation of the species name to which the gene belongs, the recognition results of our module include the prefixed species. The developed species normalization module achieved an overall F-score of 0.954 on an instance-level species normalization corpus. For gene normalization, two separate modules were respectively employed to recognize gene mentions and normalize those mentions to their Entrez Gene IDs by utilizing a multistage normalization algorithm developed for processing full-text articles. All of the developed modules are BioC-compatible .NET framework libraries and are publicly available from the NuGet gallery.

**Database URL:** https://sites.google.com/site/hjdairesearch/Projects/isn-corpus

## Introduction

Life science researchers are interested in exploring biological processes and principles, and their associated objects. Most of the biological processes are gene or protein dependent, which has spurred researchers to collect information related to genes and gene products that can assist in gaining advanced perceptions of the complex mechanisms behind biological phenomena. As a result, a large amount of biomedical literature based on gene/protein functions is published every year. Therefore, the ability to acquire timely and up-to-date information on genes/proteins cited in the large collection of biomedical literature has become a topic of interest to life scientists. To this end, data mining researchers are developing text-mining techniques to extract high-quality information from the biomedical literature. The gene/protein normalization (GN) technique (1) facilitates this process by first automatically recognizing genes and proteins mentioned in biomedical studies, and then determining their database identifiers, such as Entrez Gene IDs, to create a linkage between the literature-recorded gene/protein mentions and their corresponding biological database records. One of the major challenges encountered in GN is the disambiguation of candidate gene IDs because of the presence of orthologous genes across different species. Therefore, accurate recognition of species can provide important information that would be helpful for GN as well as many downstream tasks such as identifying protein–protein interactions (2).

The aforementioned text-mining techniques maybe developed by different research groups and used a variety of different corpora and natural language processing (NLP) modules. To facilitate the interoperability among these resources, the BioC format (3) was proposed to establish a simple XML-based format to represent, store and exchange data among different text mining systems. The collaborative BioCurator assistant task (BioC) of the BioCreative V workshop (4) attempts to integrate all state-of-the-art text mining modules into one annotation tool by providing several useful BioC-compatible NLP utilities and a BioC-encoded full text dataset. This work, which was part of the BioC task and was presented at the BioCreative V workshop, developed three BioC-compatible modules for processing abstracts and full-text articles presented in the BioC format, which can generate annotations for species and gene/protein names along with their NCBI Taxonomy IDs and Entrez Gene IDs.

Most previously released species recognition tools (5,6) only recognize complete species terms such as ‘human’ in the gene name ‘**human** brain 25 kDa alysophospholipid-specific lysophospholipase’ and normalize them to their corresponding records in the NCBI Taxonomy database. In contrast, the developed species recognition module can recognize and normalize the prefix in a gene name referring to an abbreviation of a species. For example, ‘h’ in ‘**h**LysoPLA’, and ‘Sc’ in ‘**Sc**UAP1’ should be recognized and normalized to the taxonomy IDs 9606 and 4932, respectively. To the best of our knowledge, only Ding *et al.* (7) specifically discussed this method for plant species. In addition, to facilitate the recognition and normalization of gene mentions, our multistage GN system (8) developed for processing full-text articles in BioCreative II.5 was modified to support the processing of BioC full-text articles and normalization of candidate gene mentions to their Entrez Gene IDs.

To assess the performance of entity recognition/normalization modules, a gold standard corpus in which all entities are manually annotated and linked to records in a target database is required. This work extends our instance-level GN (IGN) corpus (9) by adding annotations of species with the goal of assisting the GN system in normalizing gene mentions. Annotations of our instance-level species normalization (ISN) corpus include ordinary species terms and the prefix in a gene mention that indicate the species to which the gene belongs. The corpus is represented in the BioC XML format and is publicly available at https://sites.google.com/site/hjdairesearch/Projects/isn-corpus.

## Materials and methods

### Overview of the developed modules

**Fig. 1.**
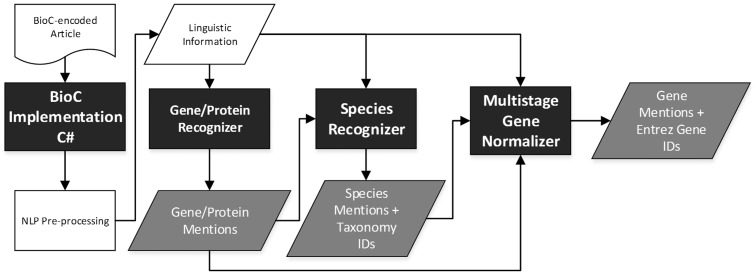
Workflow of the developed modules.

The workflow of the released modules is shown in Figure 1, in which the modules highlighted with a black background color were developed for this work. Initially, the BioC-C# implementation ported from BioC-Java implementation^1^ is used to read an article in BioC. In order to allow the developed modules to make use of the characteristics of different sections recorded in the BioC article, a utility tool, BioCAsciiKeyReader, was developed to transform the BioC-XML file into a dictionary that enables BioC-compatible modules to access the article content in an arbitrary sequence based on section headings. For each passage in the selected section, the following NLP pipeline is executed: sentence breaking, tokenization, base form and part-of-speech (PoS) tagging of each token (10) and abbreviation recognition (11). Subsequently, the gene/protein mention recognizer distinguishes gene/protein mentions based on the generated linguistic information. The recognized mentions along with the linguistic information are set as the input for the developed species recognizer to identify species terms. Finally, all of the information is aggregated and set as the input of the multistage GN module to normalize all of the recognized gene/protein mentions.

### Gene/protein recognizer module

This module recognizes gene mentions in an input article, and then matches the recognized gene mentions against a lexicon consisting of gene names, their corresponding Entrez Gene IDs and name variations generated by several rules. To recognize gene mentions, our NERBio system (12) developed based on the BioCreative II gene mention recognition corpus (13) was used. To match gene mentions, we implemented an exact matching strategy for matching Entrez Gene IDs against all of the variations listed in the lexicon.

After the gene mention matching process, the following refinement process proposed in our previous work (9) was executed. First, all recognized genes with no matched Entrez Gene ID are excluded from the output. The names of all successfully matched gene mentions are collected to form a dictionary. Finally, the exact matching algorithm is employed to search through the entire article for mentions listed in this dictionary that were overlooked by the recognizer.

### Species recognizer module

*Full* *species* *name* *recognition.* This module recognizes species information from the input article by exploiting linguistic and gene mention information. The module scans the entire article using a partial matching of species terms listed in the species dictionary created by Pafilis *et al.* (6). The species dictionary is updated by adding the base form of each species term, which was generated by using the GENIATagger (10). In addition, to support the downstream GN module in normalizing human genes, general terms such as ‘children’ and ‘patients’, which can be used as evidence to certify the co-occurring gene as a human gene, and genus names, which are normalized to their most frequently mentioned member species, are included in the dictionary. In our implementation, these terms are only recognized when they occur with at least one gene mention in the same sentence.

When processing a given article, the base forms of the full names listed as the full name-abbreviation pairs found in the article are matched before performing the actual species recognition process. If the full name is considered to be a species term, both the name and its abbreviation are added to the species dictionary for the one-off matching of the given article. Otherwise, the pairs are blacklisted for the current article. The module then performs matching against the extended dictionary. This step enables the developed module to recognize abbreviated terms defined by the authors, such as the species term ‘AMV’ in the sentence ‘The myb gene is the transforming oncogene of the avian myeloblastosis virus (AMV)’.

During the matching process, the module follows the concept of our multistage GN algorithm (8) to scan the entire article from the information-richest to the -poorest parts. Generally, most of the context knowledge, such as the full name-abbreviation information, is found in the Introduction section. In contrast, titles including the article and subsection titles are parts of the article with the least information. Therefore, the module begins scanning the article from the Introduction section if the given article is a full text, or from the abstract if the article is only an abstract. This processing order enables the module to authenticate ambiguous species terms matched with more than one taxonomic ID by selecting the ID that has been successfully linked to previously mentioned species terms—the majority rule. For example, *Escherichia coli* (taxonomy ID: 562) is usually abbreviated *E. coli* (which can be normalized to taxonomy ID: 562 or 110766). The authors may mention *Escherichia coli* in the Abstract/Introduction section with or without defining its abbreviated term *E. coli* used in the title or other sections. When processing the article using the proposed order, the module can refer to the successfully normalized ID 562 and normalize all existing *E. coli* mentions to the same ID. If the species term remains ambiguous, a predefined ID preference manually determined by our annotators is used to select the ID that is best known as a model organism.

After identifying all of the species term candidates, the PoS and the recognized gene mention information are used to filter out false-positive (FP) cases. Recognized candidates with a PoS as a verb or the boundary of which fully overlaps with another gene mention are removed.

*Prefixed* *species* *recognition*. Authors often use a designated symbol prefixed in a gene name to indicate the presence of a species. For example, an author may prefix a gene name with ‘m’ to indicate a *Mus musculus* gene, or use an abbreviated symbol containing an initial letter of the genus followed by the species name, such as ‘At’ for *Arabidopsis thaliana*. We herein refer to such symbol as ‘prefixed species’ or ‘prefix’ for simplicity. In order to recognize the prefixes, two output results associated with the given article generated by the preceding modules are used. One is the recognition results of the gene/protein recognizer module, and the other is the full name-abbreviation results generated during NLP pre-processing.

Initially, the species recognizer module matches prefixes of each recognized gene mention with an organism code list. The list consists of 1- and 2-letter organism codes manually compiled by our in-lab biologists. The 3- and 4-letter codes, such as ‘ath’ for 3702, were collected from the KEGG organism website. Furthermore, each of the full names in the complied code list was set as a query for Acromine (14), a website that provides acronym definitions observed in MEDLINE, to determine the acronym used. All of obtained definitions with length *n* were added to the corresponding *n*-letter organism code lists. Table 1 lists examples of the collected symbols.

**Table 1:** Examples of prefixed species

Symbol	Taxonomy ID	Full name
H	9606	human, *Homo sapiens*
Zm	381124	*Zea mays*
hum, hsa	9606	human, *Homo sapiens*
Ath	3701	*Arabidopsis thaliana*

Following the same matching order introduced in the previous section, the matching process begins with the information-richest section by checking prefixes of each gene name. A match is found if the prefix is listed in the organism code list followed by an uppercase letter in a gene name. If the matched gene name contains only one character after removing the prefixed symbol, then the prefix is considered to be an FP and is ignored. Alternatively, the module checks whether a full name was defined for the matched gene mention. If there is no full name, the matched prefix and the corresponding Taxonomy ID are output. Otherwise, the observed full name is further matched with the complete species names represented by the prefix. If the full name matches the species name, the prefix and its corresponding ID are obtained, or else the gene mention is blacklisted for the following prefixed species matching.

### Multistage gene normalizer module

This module uses a multistage algorithm (8) specifically developed for processing full-text articles to normalize recognized genes in a given article to their corresponding Entrez Gene IDs. The algorithm was developed based on the unique distribution of gene/protein-related information among different sections of an article. For example, the Introduction section often contains recurring information throughout the article (key genes), while normalizing a gene mention from the Results section may require resolving an acronym to the full name or associated species, which was only mentioned earlier in the Introduction section. The algorithm is divided into the following three stages.

During the first stage, GN is executed on sections with abundant information in the following order: Introduction, Discussion and Abstract. Successfully normalized IDs are kept in memory to aid GN of subsequent sections. By following the specified order, certain disambiguation rules, such as the majority rule described in the previous subsection, are more effective. Consider the majority rule as an example. The GN module aggregates all normalized IDs before processing an ambiguous gene mention with more than one matched Entrez Gene ID. Of all the ambiguous IDs aggregated, the rule selects the one with the highest frequency as the disambiguated ID. If the article were processed in its structural order, which starts with the title and ends with the Discussion and Conclusion sections, the rule would be inefficient in normalizing genes mentioned in the title due to the lack of experience of successfully matched instances. Limited contextual information in the title also leads to the failure of other disambiguation rules.

In the second stage, all normalized gene mentions and their corresponding IDs are collected to generate a dictionary. A dictionary-based tagger is executed to search the entire article for listed mentions. The tagger also examines the surrounding species information generated by the species recognizer. If a species term candidate is found and matched with a corresponding ID, then the specific ID is assigned. Otherwise, all of the ambiguous IDs matched are assigned to the gene mention. This assignment can boost the processing speed of the multistage GN, since it does not need to repeat the matching and normalization process when confronting the same gene mention again. However, the dictionary-based tagger may generate a boundary that is inconsistent with the one identified by the gene/protein recognizer. Under this circumstance, if the normalized ID is determined, then this ID is set to the recognized gene mention. Otherwise, both boundaries are reserved for later processing in the final stage.

The multistage GN module processes the remaining article sections in the final stage. Since overlapping gene mentions may be generated in the second stage, all gene mentions that have not been associated with candidate IDs are normalized and compared to overlapping ones. In the end, normalized genes with the longest span are reserved.

## Results

### Instance-level species normalization corpus

**Fig. 2.**
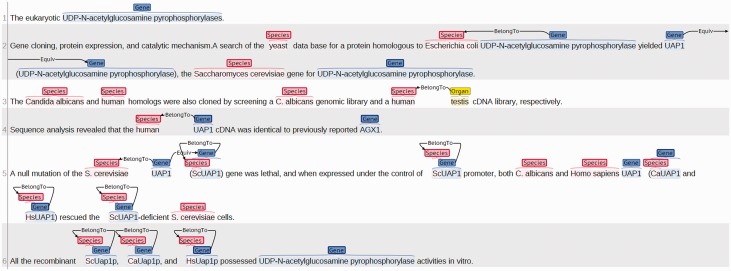
Annotations for the article PMID: 9603950.

During the preparation of this manuscript, the official gold BioC full-text corpus used in the BioCreative 2015 BioC track had not yet been released. Hence, the IGN corpus compiled in our previous work was selected for evaluation. The original IGN corpus contains instance-level annotations for human genes. In order to assess the performance of the developed species recognizer module, the corpus was extended to include annotations of species mentions. All 543 abstracts of the corpus were assigned to our in-lab annotators with life science backgrounds to annotate all mentions of species and their NCBI Taxonomy IDs. In addition, prefixes in a gene name referring to a species were also annotated. For instance, the organism symbols ‘m’ and ‘Ca’ for the gene mentions ‘**m**RPTP mu’ and ‘**Ca**Uap1p’ were annotated, which respectively represent the species ‘*Mus musculus’* and ‘*Coleophora albicans’*. The prefixes account for 13.9% of all annotations in the ISN corpus. Figure 2 shows an example of our annotations. Details of the compiled ISN corpus including the inter-annotator agreement (Fleiss’ kappa value 0.839), the distribution of the annotated species, and the number of the annotated full/prefixed species symbols are described in the online supplementary material.

*Annotation guidelines*. In order to construct the ISN corpus, our annotators were asked to annotate all substrings referring to a taxon with the aim of assisting GN systems to normalize gene mentions. The brat annotation tool (15) was used to compile the corpus. The generated annotations cover the prefixed species in gene mentions and all of the complete species strings and substrings corresponding to any taxonomic level (e.g. kingdom, phylum or division, class, order, family, genus and species) if they meet the following criteria. The complete annotation guidelines can be accessed in the online supplementary material.

The annotated species terms should include Linnaean binomial names^2^, common names and other synonyms (e.g. man and human), strain names, author-defined abbreviated words for species terms and prefixes in gene mentions referring to a species.

**Fig. 3.**
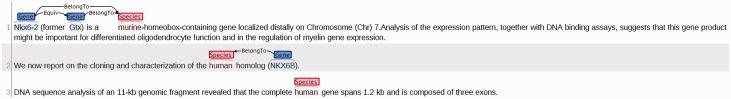
Annotations for the article PMID: 11210186.

All substrings referring to a taxon must be annotated and normalized to an NCBI Taxonomy database ID at the ‘species’ rank. These include the family or genus name of a particular species if the context infers the actual species. As shown in Figure 3, the Nkx6-2 gene belongs to the Muridae (murine) family consisting of both the house mouse (*Mus musculus*) and rat (*Rattus norvegicus*). By taking the gene’s location, chromosome 7, into consideration, the annotators can assign the species ID 10090 (mouse) to the term ‘murine’. If the substring itself or the context does not provide an implicit or explicit indication of the substring’s species, then the ID of a well-known model organism should be assigned to the substring if applicable. For example, *Drosophila* should be annotated with the taxonomy ID 7227 that indicates *Drosophila melanogaster*. Moreover, general terms such as ‘boy’ and ‘patients’ should be annotated with 9606 if the co-occurring gene mention is a human gene.

Taxonomic mentions that do not correspond to an existing NCBI Taxonomy database entry, or are misspellings, or typographic or other errors in a species term are included in the annotations if they meet the criteria stated above.

### Evaluation of species recognition performance

The performance of the developed gene/protein recognizer and multistage GN modules was reported in our previous work (8, 12, 16). Therefore, in the following experiments and discussion, we focus on evaluating the performance of the species recognizer module in terms of the standard evaluation schema: precision (P), recall (R) and F-measure (F), i.e. PRF scores, and compare its performance with two state-of-the-art species recognition tools, LINNAEUS and SPECIES.

Species recognition performance with the ISN corpus. 

Following the BioC solution proposed for biomedical text processing, the developed species recognizer module was cascaded with different modules to evaluate its performance with the ISN corpus. The exact-match criterion was used as the matching method for evaluation when calculating PRF scores at the instance level. Based on this criterion, a recognized entity is regarded as a match only if both its boundary and normalized ID fully coincide with the gold annotation.

In Table 2, the species recognizer (SR) configuration shows the stand-alone performance of the SR module without cascading with any other modules. The SR + NLP configuration combines the SR and NLP modules, including the GENIATagger and the abbreviation definition recognition software released by BioText^3^. The third configuration further uses the gene recognition results from the developed gene/protein recognizer module (G/P). The developed species recognizer module supports the sentence processing mode, which processes the given article line-by-line using no article-specific information. Its performance is listed as the fourth configuration. Finally, the performances of two state-of-the-art species recognition tools are listed in the last two rows for reference.

**Table 2:** Performance of the species recognizer module on the ISN corpus


Configuration	Recognition			Normalization		
	P	R	F	P	R	F
*Training* *set*						
1. SR	0.968	0.869	0.916	0.968	0.862	0.912
2. NLP+SR	0.962	0.874	0.916	0.962	0.868	0.912
3. NLP+G/P+SR	**0.982**	**0.958**	**0.970**	**0.982**	**0.951**	**0.966**
4. NLP+G/P+ SR-Sentence	0.970	0.949	0.960	0.968	0.940	0.953
LINNAEUS	0.970	0.811	0.884	0.946	0.785	0.858
SPECIES	0.932	0.839	0.883	0.932	0.832	0.880
* Test* *set*						
1. SR	0.963	0.820	0.886	0.957	0.814	0.880
2. NLP+SR	0.966	0.823	0.889	**0.961**	0.817	0.883
3. NLP+G/P+SR	0.965	**0.929**	**0.947**	0.960	**0.920**	**0.940**
4. NLP+G/P+SR-Sentence	**0.966**	0.917	0.941	0.952	0.900	0.925
LINNAEUS	0.951	0.764	0.847	0.918	0.734	0.816
SPECIES	0.921	0.8	0.856	0.919	0.795	0.852

The best PRF-scores are highlighted in bold. P, precision; R, recall; F, F-measure; NLP, natural language processing; G/P, gene/protein recognized module; SR, species recognizer module.

The results demonstrate that the performance of our species recognizer module can benefit from integration within the BioC-pipeline. The NLP + G/P + SR configuration achieved the best recall because it can use the information of recognized gene mentions to identify prefixed species. A comparison between the last two configurations illustrates the advantage of the article-level processing model (configuration 3) over the sentence processing model (configuration 4), as the former uses the processing order and the article-specific black/white lists to improve its performance.

An error analysis indicated that some FPs for recognizing complete species terms resulted from the process of generating species term variations. For instance, the term ‘kcat’ was recognized and assigned the taxonomy ID 9685 referring to ‘Korat cats’ because the variation ‘kcat’ was generated by removing the space from the abbreviated term ‘K cat’. The ambiguity between gene and species names is another source of FPs. For example, consider the sentence ‘Three genes of the **Popeye** (**POP**) family were detected in human, mouse and two in chicken’ of the article PMID 10882522. The gene family name ‘Popeye’ is listed as a common name for *Pleuronichthys coenosus* (Taxonomy ID: 269452). However, in the configuration of our BioC-pipeline, the upstream gene/protein recognizer module could not recognize ‘Popeye’ as a gene family name, therefore our species recognizer considered it to be a species term.

To recognize prefixed species mentioned within genes, we observed that some prefixes were recognized frequently incorrect as a species term, such as ‘Ca’ which stands for ‘calcium’ in ‘CaMKII’ and ‘CaBP1’. Different naming styles for representing those prefixes also lead to false negatives (FNs). For example, the prefixes HSA and MMU in ‘(**HSA**)SEMA6A-1’ and ‘(**MMU**)Sema6A-1’ indicate human and murine, respectively.

*Comparison of the* *species* *recognition* *performance on the LINNAEUS and Species-800* *corpora.*

In addition to the ISN corpus, we evaluated the performance of the developed module on two other corpora annotated with species information. The first is the most commonly used species corpus, the Linnaeus corpus (5), which consists of 100 full-text papers randomly selected from PubMed Central (PMC). The other is the Species-800 corpus (6), which contains 800 abstracts selected from different journals. The characteristics and differences between the two corpora and our ISN corpus are summarized as follows. (1) Both Species-800 and ISN corpora consist of abstracts, while documents in the Linnaeus corpus are full-text papers; therefore it contains some general species terms with a high degree of repetition in certain papers. For example, the general term ‘patient(s)’ accounts for 38.6%, which is even larger than the common name ‘human(s)’ (23.8%). In contrast, the Species-800 corpus does not include those general terms in their annotations, while the ISN corpus only annotates those terms when they co-occur with gene mentions. (2) The ISN corpus contains 543 articles and 2251 annotations. The Species-800 corpus contains the most articles (800) with 3708 annotations. Although the Linnaeus includes only 100 articles, it has the most annotations (4260) because of its full text-nature. The ISN corpus has the lowest diversity of species, and the Species-800 corpus contains the most diverse species. ISN corpus is the smallest, but is the only corpus that contains prefixed species annotations.

As both the Linnaeus and Species-800 corpora only contain annotations for full species names, the developed species recognition module was configured to only recognize full species names. Table 3 compares the performance of the developed species recognizer module with the performances reported in (6) for LINNAEUS and SPECIES on the two corpora at the instance level.

**Table 3:** Comparison of the species recognition performance of the developed module with LINNAEUS and SPECIES on the Linnaeus and Species-800 corpora

Corpus	Tool	Normalization		
		P	R	F
Linnaeus	LINNAEUS	0.887	0.818	0.851
	SPECIES	**0.915**	**0.908**	**0.911**
	Our module	0.892	0.728	0.802
Species-800	LINNAEUS	**0.843**	**0.754**	**0.796**
	SPECIES	0.839	0.726	0.778
	Our module	0.775	0.748	0.761

P, precision; R, recall; F, F-measure.

As shown in Table 3, the three tools exhibited different behaviors on the two corpora. We believe that this phenomenon is due to differences in the annotation standards followed by the three corpora. For the Linnaeus corpus, we observed that ‘human’ species accounted for 74.4% of all FN cases of our module. These FNs consisted of general terms like ‘participants’, ‘people’ and ‘patients’. As mentioned above, the Linnaeus corpus contains a high degree of usage of general human terms. Nevertheless, our module was developed for annotation guidelines of the ISN corpus for which annotators only annotate general human terms if they imply that the co-occurring gene mention is a human gene. Therefore, our module outputs these terms as species terms only when they co-occur with gene mentions, leading to a lower recall by our module on the Linnaeus corpus. SPECIES achieved the best performance on the corpus because Pafilis, *et al.* (6) used this corpus when developing SPECIES. The Linnaeus corpus has a filtered version that does not include species clue annotations like ‘patient’, ‘boys’, ‘murine’ and ‘chick’. Our module can omit such general terms without cascading it with G/P (the NLP + SR configuration shown in Table 2). The configuration achieved PRF-scores of 0.809, 0.909 and 0.856 respectively, for the filtered version.

On the other hand, the annotation guidelines of the ISN corpus state that the genus substring should be annotated with its well-known model organism if the context does not provide an indication of the actual species. Therefore, our species recognizer module was designed to normalize those mentions to their most common species when applicable. For instance, the Taxonomy ID 7227 (*Drosophila melanogaster*) is assigned to the generic term *Drosophila*. However, annotations of the Species-800 corpus neither include those taxon terms, such as frog and *Arabidopsis* nor general human terms observed in the Linnaeus corpus. Both circumstances, along with general English terms such as ‘white’ recognized by our module, were the main cause of FPs. Through a detailed examination of the Species-800 corpus, we observed some annotation errors in the boundaries of species terms and their normalized IDs. For example, ID 4081 for tomato was incorrectly assigned to potato (4113) in an article (PMID 20955222), and the ID for baker's yeast (4932) was incorrectly assigned to fission yeast (4896) and all other existing yeasts in another article (PMID 21600998). All such errors were reported to the corpus creator.

*Comparison of the* *effects of* *different* *species* *recognition* *tools for* *gene* *normalization.*

One of the primary goals of the developed species recognizer module is to facilitate the GN process. Therefore, we conducted an experiment to compare the effects of the species recognition results generated by the three species recognition tools for the GN task. To this end, an independent corpus, the DECA corpus (17), was selected, which contains instance-level annotations for gene mentions and their corresponding taxonomy IDs. The corpus was preprocessed to identify sentence boundaries. The three tools were run on each sentence to recognize species terms and their IDs. Finally, the following heuristic rules proposed in previous work (2, 8, 17) for species assignment were implemented to assign recognized species terms’ IDs to gene mention annotations provided in the DECA corpus.

1. Prefixed species: Assign the ID of the prefix in the gene mention referring to a species to that mention.
2. Species in the same sentence: Assign the ID of the species term to co-occurring gene mentions in the same sentence. When more than one species term occurs in the sentence, select the closest one’s ID to the left of the gene mention. If all species terms occur to the right of the gene mention, take the nearest one’s ID.
3. Focus species: Assign the most-often described species ID in the article if the gene mention does not fit into the above two rules. When two or more species are found with the same number of appearances, we select the one based on the distribution calculated from the ISN corpus, that is, select human followed by mouse, rat, yeast, fly and *E. coli*.

Table 4 compares the PRF-scores of the LINNAEUS, SPECIES and the developed species recognizer combined with the above rules on the DECA corpus. The results reported here ignore the ‘other’ and ‘not an entity’ annotations in the DECA corpus, which stand for non-frequent species and FP gene mentions, respectively, and do not have normalized taxonomy IDs.

**Table 4:** Comparison of the species recognizer tools’ performances on the DECA corpus

Tool	Normalization		
	P	R	F
LINNAEUS	0.668	0.521	0.585
LINNAEUS+	0.733	0.614	0.668
SPECIES	0.742	0.633	0.683
Our module	**0.789**	**0.648**	**0.712**

LINNAEUS: Run with LINNAEUS’s default species matcher and post-processor, which recognizes species terms from the 10 000 most frequently occurring species in MEDLINE.

LINNAEUS+: Run with entity type dictionary packs downloaded from http://linnaeus.sourceforge.net/. The packs contain updated dictionary files and support normalization of genus names and post-processing instructions.

By comparing the results generated by the studied tools, we observed that recognition of prefixes in gene mentions can improve the overall GN performance. Take, for example, the following sentence from the abstract (PMID 10578051):

The cDNA isolated for ***h*BACH**, when expressed in *Escherichia coli*, directed the expression of palmitoyl-CoA hydrolase activity and a 44-kDa protein immunoreactive to the anti-**BACH** antibody, which in turn neutralized the hydrolase activity.

All of the tools recognized the species term *Escherichia coli* (Taxonomy ID: 562). However, LINNAEUS, LINNAEUS+ and SPECIES do not provide an answer for the prefix ‘h’, which refers to species ‘human’ (Tax ID: 9606). Therefore, the heuristic rules normalized the two gene mentions, hBACH and BACH, to 562. Our module can recognize the prefixed symbol, which led to the two human genes being associated with the correct ID. In addition, the recall of our tool was better than that of SPECIES because our tool considers general terms like ‘patient’ as species terms when they co-occur with gene mentions. Although the benefit was not observed in the DECA corpus, we believe that recognizing those terms only when they co-occur with genes could avoid an overestimation of human species when we try to determine the focus species using Rule 3.

Finally, inclusion of normalizing genus names to their well-known species in our module was observed to improve the recall. For instance, in a PubMed abstract (1378625), the abstract has species terms like ‘rat (Taxonomy ID: 10116)’ and the genus name *Drosophila*. However, the authors only referred to *Drosophila melanogaster* using the prefixed symbol ‘Dm’ in the gene mention ‘DmERK-A’. Tools other than ours incorrectly assign genes mentioned in sentences with only the genus name *Drosophila* to 10116. Our tool considers the genus name and therefore can correctly assign those genes to the fruit fly. A comparison of the results between LINNAEUS and LINNAEUS+ also demonstrated a similar effect. Unfortunately, normalization of genus names may somewhat reduce the precision. For example, considering the sentence ‘… signal-dependent tyrosine phosphorylation of a *Drosophila* homolog of **extracellular signal-regulated kinase**’ of the same abstract, the rat gene ‘extracellular signal-regulated kinase’ was incorrectly associated with the fruit fly because of the genus name *Drosophila*. We believe that the problem could be addressed by formulating the species assignment task as a machine learning-based classification task that classifies the gene mention into one of the classes given its surrounding context, where each class corresponds to a species ID.

### Availability

All of the modules are implemented using the .NET framework. Table 5 provides download links for developed modules and the ISN corpus. The released tools include Microsoft .NET C# implementations of the described modules. The ISN corpus is available in the BioC format.

**Table 5:** Download links for the developed BioC-compatible modules

Resource description	Download link
BioC-C# Implementation	https://www.nuget.org/packages/NTTU.BigODM.Bio.BioC
Species Recognizer	https://www.nuget.org/packages/NTTU.BigODM.Bio.NER.Species/
Gene/Protein Recognizer/Normalizer	https://www.nuget.org/packages/IASL-BioTextMining.NamedEntityRecognition/
Instance-level Species Normalization Corpus	https://sites.google.com/site/hjdairesearch/Projects/isn-corpus

## Conclusions

In this study, we developed three modules to process articles represented in the BioC format; these modules can recognize species and gene/protein names along with their corresponding NCBI Taxonomy and Entrez Gene IDs. Our work complements the BioC project by providing Microsoft .NET framework implementation of the official BioC toolset and the three BioC-compatible .NET modules. These modules exploit characteristics of different sections of a paper to guide the normalization process for better results. Another unique contribution of this work is the species recognizer module, which identifies fully mentioned species terms, and the prefix in a gene name representing a species. The module utilizes disambiguation rules such as majority for species normalization, and the ID of the best-known model organism is assigned with the highest priority, if applicable. Moreover, we released the ISN corpus to evaluate the performance of the developed species recognizer module by including annotations of species mentions in the IGN corpus. The ISN corpus contains annotations for fully mentioned species terms, substrings referring to species, other author-defined acronyms and any prefixed species in gene mentions. All of the developed modules and the corpus are BioC-compatible and publicly available.

In the future, we would like to use the full-text dataset annotated by the BioGRID curators in the BioC track to study the distribution of various contextual information existing in the sections of full texts to enhance and provide statistical support for our multistage algorithm.

## Supplementary data

Supplementary data are available at Database Online.

Supplementary Data

## Footnotes

1 http://sourceforge.net/projects/bioc/files/BioC_Java_1.0.1.tar.gz/download.

2 A formal system of naming species of living things by giving each a name composed of two parts. For example, humans belong to the genus *Homo* and within this genus to the species *Homo sapiens*.

3 http://biotext.berkeley.edu/software.html
