# Supplementary material for: NTTMUNSW BioC modules for recognizing and normalizing species and gene/protein mentions
Source: Database (Oxford). 2016 Jul 27;2016:baw111. doi: 10.1093/database/baw111 (PMC4962763; doi:10.1093/database/baw111)
Supplement: Supplementary Data [file supp_2016_baw111_index.html]

Supplementary Data 

# NTTMUNSW BioC modules for recognizing and normalizing species and gene/protein mentions

## Supplementary Data

files

- Supplementary Data - docx file
