# Supplementary material for: NTTMUNSW BioC modules for recognizing and normalizing species and gene/protein mentions
Source: Database (Oxford). 2016 Jul 27;2016:baw111. doi: 10.1093/database/baw111 (PMC4962763; doi:10.1093/database/baw111)
Supplement: Supplementary Data [file supp_baw111_SupplementaryFile.docx]

### Instance-level Species Normalization (ISN) Corpus

The instance-level species normalization (ISN) corpus is compiled based on the instance-level gene normalization corpus^[[1]](#footnote-1)^. The ISN corpus is divided into two datasets: the training set (281 abstracts) and the test set (262 abstracts). Three in-lab annotators with a background in molecular biology annotated the corpus. A preliminary consistency test was conducted on the abstracts of the training set by having two of the annotators annotate the data, while the third checked the annotations for consistency. After examining the consistency, all annotators adjusted their annotations and re-annotated the training and test set. We measured the inner-annotator agreement between all annotations after the adjudication step using Fleiss’ kappa statistic (Fleiss, 1971).

A total of 2,251 species annotations are created. 314 annotations within 7 species (including human, house mouse, baker’s yeast, Norway rat, chicken, yellow-bellied toad and Candida albicans) are designated species symbols prefixed to gene names. Among all of the annotated prefixed symbols, human species is the most frequent one which occupies 90.4% with the following variations: “h”, “hs”, “hu”, “hum”, and “human”. Figure 1 shows the distribution of annotated species for the ISN corpus.


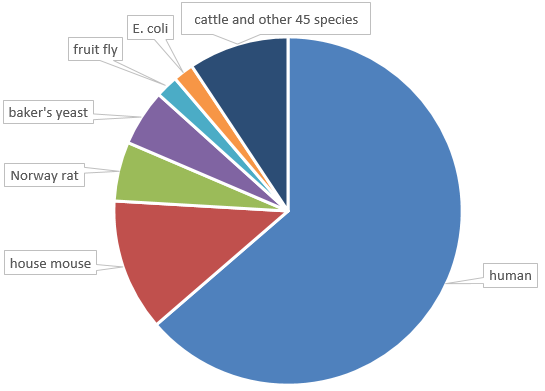


Figure 1. Species distribution of the instance-level species normalization corpus.

### ISN Corpus Annotation Guideline

Onkar Singh^1^, Hui-Jou Chou^2^, Ming-Siang Huang^3^, Hong-Jie Dai^4^

^1^Graduate Institute of Biomedical Informatics, Taipei Medical University, Taiwan, R.O.C

^2^Department of Computer science, Rutgers University-Camden, NJ, U.S.A

^3^Institute of Information Science, Academia Sinica, Taiwan, R.O.C.

^4^Department of Computer Science and Information Engineering, National Taitung University, Taiwan, R.O.C.

#### Introduction

The goal of the annotation guideline is to specify how to annotate the species terms in the instance-level species normalization (ISN) corpus.

#### Varieties of Species Annotations

The annotations cover the prefixed species symbols in gene mentions and all of the complete species strings and substrings corresponding to any taxonomic level (e.g., kingdom, phylum or division, class, order, family, genus, species) if they meet the criteria defined in the following guidelines.

##### Linnaean Binomial Name

Swedish botanist and physician Carolus Linnaeus proposed a method for naming species of living things. Their name are composed of two parts “genus and species” respectively, either parts can be in Latin grammatical forms, or they can be from other languages. For example

1. Escherichia coli (TID: 562)
2. Homo Sapiens (TID: 9606)
3. Vaccinia virus (TID: 10245)
4. Xenopus laevis (TID: 8355)

The annotated name must be normalized to their corresponding NCBI Taxonomy IDs (TIDs) as shown in the above examples.

If the authors only mentioned the genus names in the paper, those names should be annotated and normalized to the TIDs based on the context or to the most frequently mentioned member species. The annotators are suggested to refer to the records in the “dict-genera-proxy.tsv” file of “[genera-species-proxy-1.0.tar.gz](https://sourceforge.net/projects/linnaeus/files/Entity_packs/genera-species-proxy-1.0.tar.gz/download)” for the candidate TIDs.

##### Common Name

A common name of species or organism is referred as a general name which is used in a formal language of daily life, or any name that is listed in the common name record of the NCBI Taxonomy database. The following table list the examples of common names that should be considered and their corresponding TIDs.

Table 1. Common names and their corresponding IDs.

| **Common Name** | **Taxonomy ID** |
| --- | --- |
| mouse | 10090 |
| human | 9606 |
| Rat | 10116 |
| clawed frog | 8355 |

##### Abbreviated Words

Abbreviated words referred to the author-defined abbreviations for species terms. For example

- Human papilloma viruses (HPVs)
- Chinese hamster (CH): This example defines the common name of Cricetulus griseus and its abbreviated word CH.
- Saccharomyces cerevisiae (Sc)
- Candida albicans (Ca, C.albicans)

Both the full name and the abbreviated words should be annotated separately and normalized to their corresponding TIDs. Therefore, the following annotations and normalized IDs enclosed with the brackets must be created for the above examples.

- [**Human papilloma viruses**/TID: 10566] ([**HPVs**/TID: 10566])
- [**Chinese hamster**/TID: 10029] ([CH/ TID: 10029])
- [**Saccharomyces cerevisiae**/TID: 4932] ([**Sc**/TID: 4932])
- [**Candida albicans**/TID: 5476] ([**Ca**/TID: 5476], [**C.albicans**/TID: 5476])

##### Prefixed Species Symbol

The designated symbol prefixed in a gene name, which indicates the presence of a species muse be annotated. For example, the following annotations enclosed with the brackets should be created.

- [**h**]FSH-R (TID: 9606)
- [**Sc**]UAP1 (TID: 4932)
- [**Ca**]UAP1 (TID: 5476)

##### Context-specific Annotations

General terms such as “boy” and “patients” should be annotated and normalized to TID 9606, as they imply that the co-occurring gene mention is a human gene. Table 2 illustrate two cases when the annotations should be created.

Table 2. Examples for context-specific annotations

| **Example Sentence** | **Annotations** |
| --- | --- |
| The patients failed to express normal levels of beta c as shown by flow cytometry. | N/A |
| Lack of expression of the *fragile X mental retardation protein* (*FMRP*) results in mental retardation and macroorchidism, seen as the major pathological symptoms in fragile X **patients**. | patients (TID: 9606) |

The term “patients” in the second example of Table 2 should be annotated because the sentence included the protein mention “fragile X mental retardation protein”.

#### Annotation Tool and Released Format

The brat annotation tool (Stenetorp et al., 2012) was used to compile the corpus. The results of the brat annotation files were converted to the BioC format (Comeau et al., 2013). In the converted BioC file, a reference to a species term is represented by the <annotation> tag as shown in Figure 2. The text of the abstract (PMID 10022127) is shown in the <text> tag. There is one annotation for the text, which is represented in the <annotation> tag. The <text> of the <annotation> tag refers to the text (human) of the annotation. The <location> tag indicates the offset in the abstract. In this case, the species term is at the 204 characters including white spaces. The <infon> tag with the key "OrganismID" indicates the taxonomy ID for the annotation.

| <document>     <id>10022127</id>     <passage>       <infon key="type">abstract</infon>       <offset>0</offset>       <text> TIF1gamma, a novel member of the transcriptional intermediary factor 1 family.We report the cloning and characterization of a novel member of the Transcriptional Intermediary Factor 1 (TIF1) gene family, human TIF1gamma. Similar to TIF1alpha and TIF1beta, the structure of TIF1beta is characterized by multiple domains: RING finger, B boxes, Coiled coil, PHD/TTC, and bromodomain. Although structurally related to TIF1alpha and TIF1beta, TIF1gamma presents several functional differences. In contrast to TIF1alpha, but like TIF1beta, TIF1 does not interact with nuclear receptors in yeast two-hybrid or GST pull-down assays and does not interfere with retinoic acid response in transfected mammalian cells. Whereas TIF1alpha and TIF1beta were previously found to interact with the KRAB silencing domain of KOX1 and with the HP1alpha, MODI (HP1beta) and MOD2 (HP1gamma) heterochromatinic proteins, suggesting that they may participate in a complex involved in heterochromatin-induced gene repression, TIF1gamma does not interact with either the KRAB domain of KOX1 or the HP1 proteins. Nevertheless, TIF1gamma, like TIF1alpha and TIF1beta, exhibits a strong silencing activity when tethered to a promoter. Since deletion of a novel motif unique to the three TIF1 proteins, called TIF1 signature sequence (TSS), abrogates transcriptional repression by TIF1gamma, this motif likely participates in TIF1 dependent repression.</text>       <annotation id="1">         <infon key="OrganismID">9606</infon>         <infon key="type">Organism</infon>         <location offset="204" length="5"></location>         <text>human</text>       </annotation>     </passage>   </document> |
| --- |

Figure 1. BioC Annotation Format

### References

Comeau, D. C., Doğan, R. I., Ciccarese, P., Cohen, K. B., Krallinger, M., Leitner, F., . . . Wilbur, W. J. (2013). BIoC: A Minimalist Approach to Interoperability for Biomedical Text Processing. *Database: the journal of biological databases and curation*.

Fleiss, J. L. (1971). Measuring nominal scale agreement among many raters. *Psychological bulletin, 76*(5), 378-382.

Stenetorp, P., Pyysalo, S., Topić, G., Ohta, T., Ananiadou, S., & Tsujii, J. i. (2012). *brat: a Web-based Tool for NLP-Assisted Text Annotation*. Paper presented at the Proceedings of the Demonstrations Session at EACL 2012, France.

1. <https://sites.google.com/site/hongjiedai/projects/the-ign-corpus> [↑](#footnote-ref-1)
